# Preliminary Identification of Putative Terpene Synthase Genes in *Caryocar brasiliense* and Chemical Analysis of Major Components in the Fruit Exocarp

**DOI:** 10.3390/life16010067

**Published:** 2026-01-01

**Authors:** Helena Trindade, Bruno Nevado, Raquel Linhares Bello de Araújo, Viviane Dias Medeiros Silva, Lara Louzada Aguiar, Ana Ribeiro, Julio Onesio-Ferreira Melo, Paula Batista-Santos

**Affiliations:** 1 Centre for Ecology, Evolution and Environmental Change (CE3C), Global Change and Sustainability Institute, (CHANGE), Faculty of Sciences, University of Lisbon (FCUL), 1749-016 Lisbon, Portugal; htrindade@fc.ul.pt (H.T.); bnevado@ciencias.ulisboa.pt (B.N.); 2 Departamento de Alimentos, Faculdade de Farmácia, Campus Belo Horizonte, Universidade Federal de Minas Gerais, Belo Horizonte 31270-901, MG, Brazil; raquellba.linhares@gmail.com (R.L.B.d.A.); vivianedms05@gmail.com (V.D.M.S.); laralaguiar@hotmail.com (L.L.A.); 3 Departamento de Ciências Exatas e Biológicas, Universidade Federal de São João del-Rei (UFSJ), Campus Sete Lagoas, Sete Lagoas 35701-970, MG, Brazil; 4 LEAF—Instituto Superior de Agronomia, Universidade de Lisboa, Tapada da Ajuda, 1349-017 Lisboa, Portugal; acribeiro@ff.ulisboa.pt; 5 Faculdade Farmácia, Universidade de Lisboa, Av. Prof. Gama Pinto, 1649-003 Lisboa, Portugal; 6 Centro de Estudos Florestais, Laboratório Associado TERRA, Instituto Superior de Agronomia, Universidade de Lisboa, Tapada da Ajuda, 1349-017 Lisboa, Portugal

**Keywords:** bioactive compounds, Brazilian savanna, genomics, pequi, phylogenetics, tepene synthases

## Abstract

**Background:** *Caryocar brasiliense* Camb. Caryocaraceae is a typical tree from the Brazilian Cerrado with commercial importance due to its edible fruit, known as pequi. This native plant holds significant economic value and is a key candidate for cropping systems. Rich in phytochemicals, such as phenolics, flavonoids, and terpenoids, it has shown notable health benefits. **Methods:** Considering the importance of terpenes and their biological properties, and based on the first draft genome of *C. brasiliense*, this study aimed to identify putative terpene synthase genes and classify them into the phylogenetic subfamilies previously identified across all plant lineages. The presence of terpenes was also verified in samples of the outer portion of the fruit by solid-phase microextraction gas chromatography mass-spectrometry. **Results:** Analysis of genome completeness showed that over 90% of genes were identified despite a highly fragmented assembly, with 71% containing complete gene sequences. Twenty-two genes were retained as putative terpene synthase genes considering their homology with the terpene synthase Hidden Markov Model (HMM) profiles in the Pfam-A database. Ten sequences with a minimum length of 298 amino acids were used for phylogenetic inference. In the resulting phylogenetic tree, *C. brasiliense* terpene synthase genes clustered within the different previously identified Angiosperm clades and allowed us to classify each gene into different phylogenetic subfamilies: six genes belonged to the h/d/a/b/g, three to the c, and one to the e/f. The headspace solid-phase microextraction technique, in conjunction with gas chromatography mass-spectrometry, has allowed for the identification of eleven chemical compounds, including a terpene. **Conclusions**: This initial identification of putative terpene synthase genes in pequi, together with the chemical analysis of the outer fruits, lays the groundwork for future studies aimed at optimizing terpene biosynthesis for both biological and commercial applications.

## 1. Introduction

The Cerrado biome is the second-largest biome of Brazil and is considered a hotspot due to the rapid loss of biological diversity caused by agriculture, livestock, and urbanization [1]. Pequi (*Caryocar brasiliense* Camb.) is a typical edible fruit from the Brazilian Cerrado, with great occurrence and economic importance in this region. Pequi trees play a valuable ecological role in these ecosystems, since they are a source of food and habitat for fauna [2]. These fruits are rich in nutrients, have unique sensory characteristics, and are used in regional cuisine, for the preparation of flours, for the extraction of oils for cosmetics, and for their therapeutic properties [3,4,5].

Pequi has recently been reported to have analgesic and anti-inflammatory properties [6], reducing oxidative stress, inflammation, and anemia associated with aging in Swiss mice [7]. It is also referred to have anticholinesterase and antioxidant activities, as well as to prevent memory loss in mice caused by aluminum consumption and brain lipid peroxidation [8]. Other studies with the ethanolic extract of pequi bark have shown very low toxicity in vitro and in vivo [9,10] as well as a protective effect against oxidative stress in human coronary artery endothelial cells [11], supporting its medicinal potential.

The compounds associated with positive health impacts include a list of specialized metabolites with active principles, ranging from phenolics, terpenes, and alkaloids [12,13]. Terpenoids are the largest group of specialized metabolites, and tens of thousands of terpenoid compounds have been identified in higher plants [14]. Chemically, they are polymeric isoprene units (C5) that can be arranged in various lengths of backbone polymers. Terpene synthase enzymes can give rise to a multitude of molecules that have been pivotal to the survival and evolution of higher plants. Furthermore, these compounds have been associated with beneficial health effects, such as anti-aggregatory, antiallergic, anti-coagulation, anti-inflammatory, neuroprotective, sedative, analgesic [15], and other biological properties [16], including antimicrobial and antifungal activities [15,17]. Some terpenes can be considered as ecological pesticides, and essential oils rich in terpenes have proven activity against several fungi [18,19]. Studies based on individual terpenes have also shown fungicide activity against *Botrytis cinerea*, a plant pathogen that affects cultures [20]. Terpenes have been previously identified from pequi fruits [21,22,23] and include several monoterpenes, e.g., *α*-phellandrene, *β*-myrcene, *β*-ocimene, and the diterpene geranyllinalool, just to mention a few. The technique of headspace solid-phase microextraction with gas chromatography—mass spectrometry analysis has been used to identify terpenes and other volatile compounds present in different fruits of the Cerrado, such as *Eugenia dysenterica* [24], *Eugenia brasiliensis* [25], *Eugenia klotzschiana* [26], and pequi peel [27]. Fruit peels have been used to extract a variety of bioactive compounds, including terpenes [28].

Considering the published genome sequencing data for *Caryocar brasiliense* [29], mining this information will allow the identification of genes responsible for important biological characteristics. In this study, we focused on the identification of putative genes involved in terpene biosynthesis in pequi, laying the foundation for future biotechnological approaches to improve terpene synthesis, several of which rely on yeast systems [30,31,32]. Our preliminary study should be extended, and functional gene characterization needs to be performed for full validation of gene function. These biotechnological tools are considered of utmost importance, considering that terpene extraction from natural sources, as performed in the past, nowadays raises environmental concerns and is no longer considered a viable option.

## 2. Materials and Methods

### 2.1. Sequence Retrieval and Identification of Putative Terpene Synthase Genes

In this study, genomic sequences from *C. brasiliense* [29] were obtained from GenBank under accession number GCA_004918865.1 (Table 1). To infer the genome completeness, BUSCO v. 4.1 (Benchmarking Universal Single-Copy Orthologs) [33] with the Viridiplantae database was used. To identify the Terpene synthase genes, the genome of *C. brasiliense* was annotated using the MAKER pipeline v2.31 [34]. Both ab initio and homology-based evidence were used and obtained from the proteomes of related species in the Malpighiales order (Table 2), available from the 1KP database [35]. The resulting protein-coding genes against the Pfam-A database were searched using interproscan v 5.61 [36]. We classified as putative Terpene-synthase genes all genes with the best hit against the Terpene_synth_C (PF03936) or the Terpene synthase N-terminal domain (PF01397) profiles [37].

### 2.2. Phylogenetic Analyses

To classify the putative Terpene synthase genes of *C. brasiliense* into the phylogenetic subfamilies identified in previous studies [37], we obtained the unaligned sequence data containing all Terpene synthase genes (longer than 350 amino acids) previously identified across green plants [37]. The newly identified Terpene synthase genes from *C. brasiliense* (minimum length: 298 amino acids) were added to this dataset. All data were aligned using mafft v 7.5 [38] with 1000 iterations of improvement. The best-fitting protein evolution model was identified with modeltest-ng v.0.1.7 [39,40]. The phylogenetic inference was performed using raxml-ng v.1.1 [41] with 10 random starting trees and 100 bootstrap replicates, using the best-fitting protein evolution model (JTT + G + F).

### 2.3. Solid-Phase Microextraction Gas Chromatography–Mass Spectrometry

Polydimethylsiloxane/Divinylbenzene (PDMS/DVB, 65 μm) fibers were employed for the solid-phase microextraction (SPME) and gas chromatography-mass spectrometry (GC-MS) analysis. Samples of the outer portion of three fruits of *C. brasiliense* weighing 1.0 g were transferred to 20 mL headspace vials, in triplicate, which were then sealed. The samples were taken to a heating plate, on which an aluminum block with a cylindrical bore was placed, in order to place the headspace vials for sample heating. Samples were pre-heated for 5 min, after which the PDMS/DVB fiber holder was inserted into the vial, and the fiber was exposed with the temperature kept at 50 °C for 10 min. The PDMS/DVB fiber was then retracted, transferred to the GC-MS injector, and exposed, where it remained in the equipment for 5 min and was retracted for the remainder of the run [42].

A gas chromatograph coupled with a mass spectrometer (Shimadzu Scientific Instruments, Kyoto, Japan). A split/splitless injector in splitless mode was used as an ion-trap type analyzer, and it was maintained for 5 min at a temperature of 250 °C. Helium gas (1 mL min^−1^ flow) was used with a HP-5, 30 m × 0.25 mm × 0.25 μm, MS capillary column (5% phenyl and 95% methylpolysiloxane) (Agilent Technologies Inc., Munich, Germany). The column was held at 40 °C for 1 min, and then, the temperature was increased at a rate of 12 °C min^−1^ up to 120 °C, maintaining it for 2 min, followed by an increase of 15 °C min^−1^ up to 150 °C and at 20 °C min^−1^ to 245 °C, held for 2 min [26].

Mass spectrometry was set to fragment ions between 35 and 300 *m*/*z* in 70 eV electron impact ionisation mode; the transference line temperature was 275 °C, and the ion source temperature was 200 °C. Volatile compounds were identified based on the mass-to-charge ratio (*m*/*z*) of the sample ion fragments corresponding to each peak generated by the chromatogram. The mass spectra of the analytes found were compared with the mass spectra data obtained from the NIST library (National Institute of Standards and Technology), using the 2011 version of the NIST/EPA/NIH Mass Spectral Database (NIST 11), using Xcalibur software version 2.1 (Thermo Scientific, San Jose, CA, USA), and considering the level of similarity (reverse lookup index, RSI) greater than 600. The RSI index consists of a numerical comparison factor where the higher its value, the closer the compound is to the finding in the NIST library literature. However, only peaks with a value above 600, a relative standard intensity (RSI) and a signal-to-noise ratio (S/N) above 50 decibels were selected.

## 3. Results and Discussion

Analysis of genome completeness using BUSCO showed that, despite a highly fragmented assembly, over 90% of BUSCO genes were found in the genome assembly of *C. brasiliense*, with 71% containing complete gene sequences and an additional 21% of genes present but fragmented (Table 1). We identified 33,767 protein-coding genes using the MAKER pipeline. Of these, 22 genes had homology with either (or both) of the Terpene synthase Pfam-A profiles and were thus retained as putative Terpene synthase genes (Table 3). Search on NCBI Conserved Domain [43] allowed us to find several motifs and domains that validated the sequences as partial putative terpene synthases. Larger sequences, CbTPS19, CbTPS20, CbTPS21, and CbTPS22, with respectively 458, 497, 561, and 729 amino acids, showed at least four (or five) of the total five conserved domains characteristic of these terpene synthases (Table 3).

**Figure 1:**
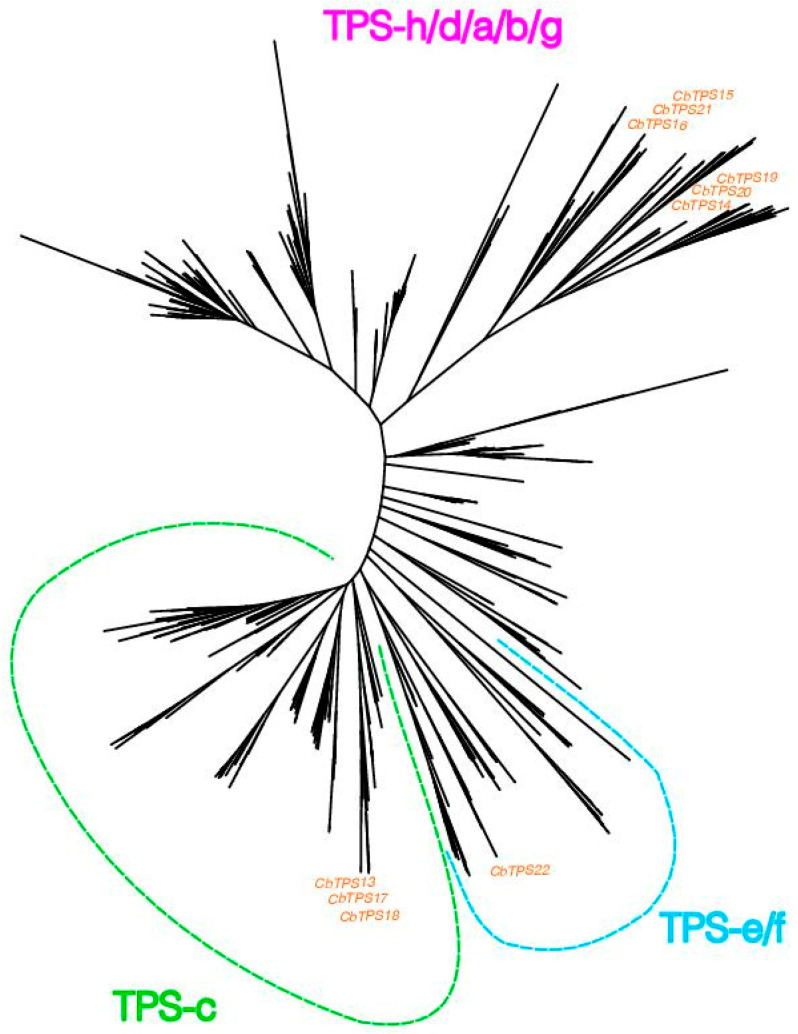
Phylogeny of Terpene synthase genes, including the ten longest genes identified in *C. brasiliense* (denoted in orange). Classification into families (TPS-e/f, TPS-c, and TPS-h/d/a/b/g) follows from figure 3 in reference [37].

Of the 22 genes identified, we used the ten longest (minimum length: 298 aa; Table 3) for phylogenetic inference. In the resulting phylogenetic tree, *C. brasiliense* terpene synthase genes clustered within the different angiosperm clades identified in previous work [37] and allowed us to classify each gene into the different phylogenetic subfamilies (Figure 1). Six genes belonged to the h/d/a/b/g subfamily: *CbTPS14*, *CbTPS19*, and *CbTPS20* clustered together with accessions from *Arabidopsis thaliana*, while *CbTPS15*, *CbTPS16*, and *CbTPS21* clustered together with accessions from *Oryza sativa*. The genes forming the group TPS-h/d/a/b/g are apparently involved in secondary (specialized) metabolism [37].

Considering the remaining four genes, three genes belonged to the c subfamily, namely *CbTPS13*, *CbTPS17*, and *CbTPS18*, and one gene, *CbTPS22*, was assigned to the e/f subfamilies. Both TPS-c and TPS-e/f subfamilies can be involved in gibberellin biosynthesis, but they can also give rise to numerous proteins involved in secondary metabolism.

The partial sequences we identified here can be used for primer design that will allow gene amplification, followed by heterologous gene expression. This will allow the obtention of protein that will be used to assay enzymatic activity. The final outcome will be the identification of the respective terpene synthases [44,45].

**Figure 2:**
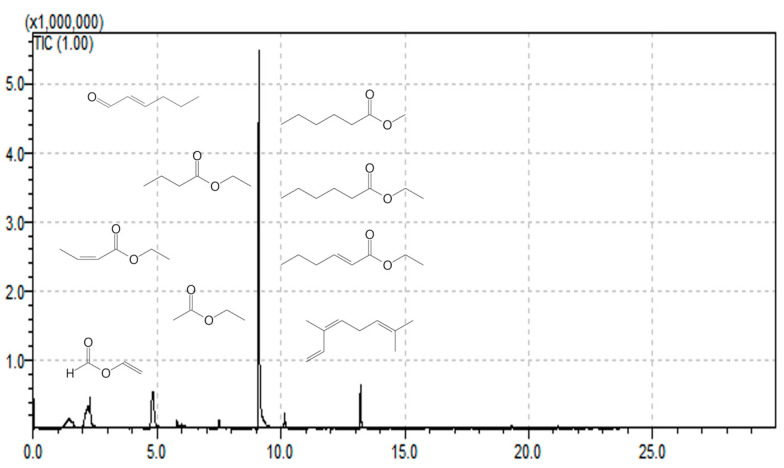
*C. brasiliense* exocarp extract chromatogram. The *Y*-axis shows abundance (AU), and the *X*-axis shows run time in minutes.

Gas chromatography-mass spectrometry analyses revealed a diverse phytochemical profile (Table 4) comprising five carboxylic acid esters (butanoic acid ethyl ester, (*E*)-2-butenoic acid ethyl ester, methyl hexanoate, ethyl hexanoate, and ethyl octanoate), one ethyl ester of carboxylic acids (all identified as ethyl acetate), one *α*-amino acid (alanine), one *α*,*β*-unsaturated aldehyde ((*E*)-2-hexenal), one *α*,*β*-unsaturated carboxylic acid ester (ethyl 2-hexenoate), one monoterpene hydrocarbon ((*Z*)-*β*-ocimene), and one formate ester (ethenyl formate). Figure 2 shows the resulting chromatogram of the HS-SPME-GC-MS analyses of samples of *C. brasiliensis*.

The *α*,*β*-unsaturated aldehyde (*E*)-2-hexenal (RT 5.995) functions as a critical green leaf volatile (GLV) rapidly synthesized via the lipoxygenase pathway following tissue damage [46]. The conjugated double bond system confers significant antimicrobial and antifungal properties, serving as part of the plant’s chemical defense arsenal against a broad spectrum of phytopathogens. Recent research has demonstrated its efficacy against economically important pathogens, including *Botrytis cinerea* and various *Colletotrichum* species, with minimum inhibitory concentrations in the low ppm range [47,48]. Agricultural applications have expanded to include (*E*)-2-hexenal-based biopesticides and plant elicitors that activate systemic acquired resistance mechanisms, potentially reducing conventional fungicide requirements by 30–40% when incorporated into integrated pest management programs [49].

Alanine (RT 1.420), an α-amino acid, plays fundamental roles in primary metabolism beyond its function as a protein building block. This compound participates centrally in transamination reactions and the alanine-glucose cycle that regulates carbon and nitrogen flux between plant tissues [50]. During environmental stress conditions, particularly drought and hypoxia, alanine accumulation serves as both a biochemical stress indicator and adaptive response, functioning as a compatible solute that maintains cellular osmotic balance without disrupting enzyme function [51].

The monoterpene hydrocarbon (*Z*)-*β*-ocimene (RT 10.150) represents a significant volatile compound derived from the isoprenoid pathway through the MEP pathway in plastids [52]. This acyclic terpene functions prominently in tritrophic plant-herbivore-predator interactions, serving as both a herbivore deterrent and an attractant for natural enemies, including parasitoid wasps and predatory insects. Studies have demonstrated that plants under herbivore attack can increase *β*-ocimene emissions by up to 1000-fold, triggering defense responses in neighboring plants through volatile signaling networks [53]. The compound’s conjugated diene structure confers notable antioxidant properties, with radical-scavenging activity comparable to vitamin E analogs in some assay systems [54]. *β*-ocimene serves as a key component of essential oil-based formulations targeting agricultural pest management, particularly in organic production systems where conventional pesticides are restricted [55].

## 4. Conclusions

In conclusion, this analysis based on the pequi draft genome allowed us to recover the majority of BUSCO genes, including most complete sequences, and to identify 22 putative terpene synthase genes. Phylogenetic analysis of ten well-supported sequences further revealed their distribution across established Angiosperm clades, enabling classification into distinct terpene synthase subfamilies and providing insight into their evolutionary relationships. These preliminary results lay the foundation for further studies to fully characterize terpene synthase gene sequences in pequi and to explore their potential applications. providing a basis for future investigations that may include quantification and functional validation. Furthermore, solid-phase microextraction gas chromatography mass-spectrometry has allowed the identification of significant chemical compounds, including a terpene that plays a key role in this species metabolism and putatively displays significant applications in the food, health, and agricultural industries.

## Figures and Tables

**Table 1:** Summary statistics of the *C. brasiliense* genome used in this study.

Statistics	
Total sequence length (bp)	212,172,521
Number of scaffolds	55,248
Scaffold N50	6005
Scaffold L50	10,533
Complete BUSCOs	302 (71.1%)
Fragmented BUSCOs	88 (20.7%)
Missing BUSCOs	35 (8.2%)

**Table 2:** Malpighiales proteomes used for genome annotation.

Species Name
*Bischofia javanica*
*Chrysobalanus icaco*
*Croton tiglium*
*Drypetes deplanchei*
*Erythroxylum coca*
*Galphimia gracilis*
*Garcinia oblongifolia*
*Hypericum perforatum*
*Licania michauxii*
*Linum bienne*
*Malesherbia fasciculata*
*Mammea americana*
*Ochna serrulata*
*Passiflora edulis*
*Rhizophora mangle*
*Salix acutifolia*
*Viola tricolor*

**Table 3:** Detailed information on each putative Terpene synthase gene, including the conserved domains and motifs found.

		Conserved Domains					Conserved Motif
Identification	Size (aa)	Active Site Lid Residues	Substrate Binding Pocket	Substrate-Mg^2+^ Binding Site	Aspartate-Rich Region 1	Aspartate-Rich Region 2	DDxxD
CbTPS01	50						
CbTPS02	52						
CbTPS03	53						
CbTPS04	57				x		X
CbTPS05	74						
CbTPS06	83						
CbTPS07	109						
CbTPS08	123					x	
CbTPS09	124						
CbTPS10	229				x		X
CbTPS11	287			x	x		X
CbTPS12	294						
CbTPS13 *	298		x	x	x		
CbTPS14 *	325		x	x	x		X
CbTPS15 *	391				x		X
CbTPS16 *	394				x		X
CbTPS17 *	443				x		
CbTPS18 *	448				x		
CbTPS19 *	458		x	x	x	x	X
CbTPS20 *	497		x	x	x	x	
CbTPS21 *	561	x	x	x	x	x	X
CbTPS22 *	729		x	x	x	x	X

* genes with a minimum coding region corresponding to 298 amino acids that were used for phylogenetic analysis. DDxxD corresponds to a highly conserved aspartate-rich sequence, D: aspartate; x: any amino acid.

**Table 4:** Qualitative results of solid-phase microextraction gas chromatography-mass spectrometry of *C. brasiliensis* exocarp analysis.

Peak N.	Retention Time (min)	Compound	Formula	CAS	Chemical Class
1	1.420	Alanine	C_3_H_7_NO_2_	56-41-7	α-Amino acid
2	1.515	Formic acid, ethenyl ester	C_3_H_4_O_2_	692-45-5	Formate ester
3	2.200	Ethyl Acetate	C_4_H_8_O_2_	141-78-6	Ethyl ester of a carboxylic acid
4	4.825	Butanoic acid, ethyl ester	C_6_H_12_O_2_	105-54-4	Carboxylic acid ester
5	5.800	(*E*)-2-Butenoic acid, ethyl ester,	C_6_H_10_O_2_	623-70-1	Carboxylic acid ester
6	5.995	2-Hexenal, (E)-	C_6_H_10_O	6728-26-3	Unsaturated aldehyde
7	7.500	Hexanoic acid, methyl ester	C_7_H_14_O_2_	106-70-7	Carboxylic acid ester
8	9.105	Hexanoic acid, ethyl ester	C_8_H_16_O_2_	123-66-0	Carboxylic acid ester
9	10.100	2-Hexenoic acid, ethyl ester	C_8_H_14_O_2_	1552-67-6	*α*,*β*-Unsaturated carboxylic acid ester
10	10.150	1,3,6-Octatriene, 3,7-dimethyl-, (Z)-	C_10_H_16_	3338-55-4	Monoterpene hydrocarbon
11	13.210	Octanoic acid, ethyl ester	C_10_H_20_O_2_	106-32-1	Carboxylic acid ester

## Data Availability

The coding sequences of the Terpene synthase genes presented in this article are not readily available because of time limitations. Requests to access the datasets should be directed to the coauthors Helena Trindade (htrindade@fc.ul.pt) or Bruno Nevado (bnevado@ciencias.ulisboa.pt). The remaining Terpene sequences were kindly provided by Qidong Jia [32].
